# Beyond valgus stress radiography: arithmetic HKA angle (aHKA) as a superior predictor of limb alignment after UKA

**DOI:** 10.1186/s42836-025-00352-9

**Published:** 2025-12-15

**Authors:** Naoki Nakano, Masanori Tsubosaka, Tomoyuki Kamenaga, Yuichi Kuroda, Kazunari Ishida, Shinya Hayashi, Ryosuke Kuroda, Tomoyuki Matsumoto

**Affiliations:** 1 Department of Orthopaedic Surgery, Kobe University Graduate School of Medicine, 7-5-1 Kusunoki-Cho, Chuo-Ku, Kobe, Hyogo 650-0017 Japan; 2 Department of Orthopaedic Surgery, Kobe Kaisei Hospital, 3-11-15, Shinoharakita-Machi, Nada-Ku, Kobe, Hyogo 657-0068 Japan

**Keywords:** Unicompartmental knee arthroplasty, Coronal alignment, Arithmetic hip-knee-ankle angle, Femorotibial angle, Valgus stress radiograph

## Abstract

**Purpose:**

Accurate prediction of postoperative coronal alignment is essential for successful outcomes following medial unicompartmental knee arthroplasty (UKA). Traditionally, valgus stress femorotibial angle (FTA) has been used to estimate the correctability of varus deformity; however, its reliability is limited by dependence on soft tissue behavior and examiner technique. In contrast, the arithmetic hip–knee–ankle angle (aHKA), calculated from bony anatomy, offers an objective and reproducible measure of constitutional limb alignment. While early studies suggest aHKA correlates well with postoperative alignment, direct comparison with valgus stress FTA has been lacking. This study aimed to compare the predictive accuracy of aHKA and valgus stress FTA for postoperative alignment and alignment correction (ΔHKA) in medial UKA.

**Methods:**

This retrospective study included 125 knees undergoing medial fixed-bearing UKA. Preoperative aHKA was calculated from long-leg radiographs, and valgus stress FTA was measured using a Telos arthrometer. Postoperative hip–knee–ankle angle (HKA) was obtained from standardized radiographs. Correlation analyses were performed between postoperative HKA and both aHKA and 360°–valgus stress FTA. Similarly, correlations were assessed between ΔHKA and (aHKA–preoperative HKA) as well as (360°–valgus stress FTA–preoperative HKA). Fisher’s Z-test was used to assess differences in correlation strengths.

**Results:**

Postoperative HKA showed stronger correlation with aHKA (R^2^ = 0.5003, *P* < 0.001) than with 360°–valgus stress FTA (R^2^ = 0.1157, *P* < 0.001), with a statistically significant difference (Z = −4.12, *P* < 0.001). ΔHKA was more strongly associated with aHKA–preoperative HKA (R^2^ = 0.3805, *P* < 0.001) than with 360°–valgus stress FTA–preoperative HKA (R^2^ = 0.1111, *P* < 0.001) (Z = −2.92, *P* = 0.0036).

**Conclusion:**

aHKA demonstrated superior predictive accuracy for both postoperative alignment and alignment correction compared to valgus stress FTA. As a bone-based and examiner-independent parameter, aHKA is a valuable tool for preoperative planning in medial UKA and may reduce the need for stress radiography.

Video Abstract

**Supplementary Information:**

The online version contains supplementary material available at 10.1186/s42836-025-00352-9.

## Introduction

Unicompartmental knee arthroplasty (UKA) has become an established surgical option for selected patients with isolated compartmental osteoarthritis of the knee, offering faster recovery, better functional outcomes, and greater preservation of native joint kinematics compared to total knee arthroplasty (TKA) [1–4]. Among the many factors that influence postoperative success, restoration of appropriate coronal limb alignment is widely considered to be critical. Although a certain degree of under-correction may be permissible to maintain normal knee biomechanics and avoid overloading the contralateral compartment, excessive residual varus or valgus can predispose to early polyethylene wear, component loosening, and progression of osteoarthritis in the non-operated compartment [5–7].

Traditionally, valgus stress radiography (femorotibial angle: FTA) has been used to evaluate the correctability of preoperative varus deformity in the context of medial UKA [8–10]. By applying a manual or device-assisted valgus load during radiographic imaging, surgeons have attempted to simulate the dynamic conditions under which the knee alignment might change following surgery, providing an estimate of anticipated postoperative alignment. However, the validity and reproducibility of this method have been questioned, as it relies heavily on soft tissue behavior, patient cooperation, and the consistency of stress application [11–13]. Furthermore, inter-observer and intra-observer variability remains a concern, particularly in centers without standardized protocols or specialized equipment.

In recent years, the arithmetic hip–knee–ankle angle (aHKA) has been proposed as an alternative method for estimating constitutional limb alignment [14, 15]. Unlike valgus stress testing, aHKA is derived purely from the bony anatomy of the femur and tibia—specifically, the lateral distal femoral angle (LDFA) and the medial proximal tibial angle (MPTA). As such, it provides a theoretically objective measure that is independent of soft tissue laxity or examiner technique. aHKA has been shown to correlate strongly with postoperative alignment in medial UKA, suggesting its potential usefulness as a preoperative planning tool [16]. In addition, the difference between aHKA and preoperative HKA has been found to correlate well with the degree of alignment correction achieved through UKA (ΔHKA), indicating its utility in estimating the extent of coronal realignment achieved through UKA [17].

The principal advantage of aHKA lies in its reproducibility and resistance to the confounding effects of soft tissue condition, joint stiffness, or patient-specific variability. This makes it particularly appealing in contemporary surgical planning, where precision and objectivity are increasingly valued. Nevertheless, despite its theoretical strengths and promising early evidence, the comparative efficacy of aHKA versus traditional valgus stress FTA in predicting postoperative alignment has yet to be clearly established. Similarly, it remains to be seen whether the difference between aHKA and preoperative HKA serves as a more robust predictor of alignment change than valgus stress FTA.

Therefore, the purpose of this study is twofold. First, we aim to compare the predictive value of aHKA and valgus stress FTA in estimating postoperative limb alignment following medial UKA. Second, we seek to determine whether the difference between aHKA and preoperative HKA provides a more accurate estimate of the expected change in coronal alignment than the difference between valgus stress FTA and preoperative HKA. By addressing these questions, we hope to clarify the clinical utility of aHKA in preoperative decision-making and potentially support a shift away from reliance on stress radiographs in UKA planning.

## Materials and methods

The study protocol was approved by the Ethics Committee of Kobe University Graduate School of Medicine (Approval No. 1510, Date of Approval: 2 December 2013). This retrospective study included 101 consecutive patients (125 knees) who underwent medial fixed-bearing UKA using the Persona Partial Knee System (Zimmer Biomet Inc., Warsaw, IN, USA). Informed consent was obtained from all participants prior to surgery. Eligibility criteria for UKA comprised a radiographic diagnosis of isolated end-stage (Kellgren–Lawrence grade 4) medial compartment osteoarthritis (OA) or osteonecrosis (ON), with an active range of motion (ROM) of ≥ 90°, a fixed flexion deformity of ≤ 15°, and a varus deformity of ≤ 10°. Patients diagnosed with both ON and end-stage OA were categorized as having OA.

The mean age of patients was 74.2 ± 8.0 years (range, 51–89 years). Among the cohort, 39 knees (31.2%) (in 30 patients) were male, and 86 knees (in 71 patients) (68.8%) were female. UKA was performed on the left knee in 59 cases (47.2%) and on the right knee in 66 cases (52.8%). A total of 106 knees (84.8%) were diagnosed with OA, and 19 knees (15.2%) with ON (Table 1). Preoperative magnetic resonance imaging (MRI) was conducted in all cases. Patients lacking an intact anterior cruciate ligament (ACL) or exhibiting involvement of the lateral compartment articular surface were excluded from UKA and instead underwent total knee arthroplasty (TKA). All 125 UKA procedures were included in the analysis and were performed by two senior surgeons, each with over 15 years of experience.

**Table 1:** Patients’ characteristics

Variables	Value
Age (years)	74.2 (range, 51–89; SD: 8.0)
Gender (male:female)	30 (39 UKAs):71 (86 UKAs)
Side of UKA (left:right)	59:66
Diagnosis (OA:ON)	106:19

The values are given as the mean and standard deviation for continuous variables

UKA: unicompartmental knee arthroplasty, OA: osteoarthritis, ON: osteonecrosis, SD: standard deviation

Following inflation of the tourniquet to 250 mmHg, a limited medial parapatellar approach was undertaken. Intraoperative macroscopic assessment confirmed that the articular surfaces of both the lateral and patellofemoral compartments were intact. Minimal soft tissue release of the medial structures was performed, along with osteophyte excision. A proximal tibial osteotomy was carried out using an accelerometer-based portable navigation system (OrthAlign Plus®, UniAlign™; OrthAlign Inc., Aliso Viejo, CA, USA), which facilitates accurate tibial bone resection in UKA, with control of both coronal and sagittal alignment. According to the manufacturer’s data, this system achieves a measurement accuracy of ± 0.5° when determining the angle between the OrthAlign Plus® device and the reference sensor. Preoperative planning targeted neutral coronal alignment (0° varus). Sagittal alignment was determined with reference to the native posterior tibial slope, measured relative to a line perpendicular to the sagittal axis. The sagittal axis of the tibia was defined as a line connecting the anterior one-third of the medial tibial plateau to the midpoint of the tibial plafond. The target sagittal alignment was set at 6.0° when the native angle was ≤ 6.0°, 8.0° when ≥ 8.0°, and equal to the measured angle when it fell between 6.0° and 8.0°, in accordance with previously published protocols [18–20]. The planned resection depth of the proximal tibia, excluding the thickness of the saw blade, was set at 4 mm in all cases and was guided using a dedicated stylus. Subsequent to the tibial resection, a distal femoral osteotomy was performed using the spacer block technique, with reference to the proximal tibial cut surface. Femoral rotational alignment was meticulously adjusted to correspond with the mechanical axis of the tibia. The remaining femoral resections, i.e., posterior and chamfer cuts, were then completed. After implant placement of both the femur and tibia, the insert thickness was selected to achieve appropriate tension, allowing insertion of a 2-mm tension gauge but not a 3-mm one in extension.

**Fig. 1:**
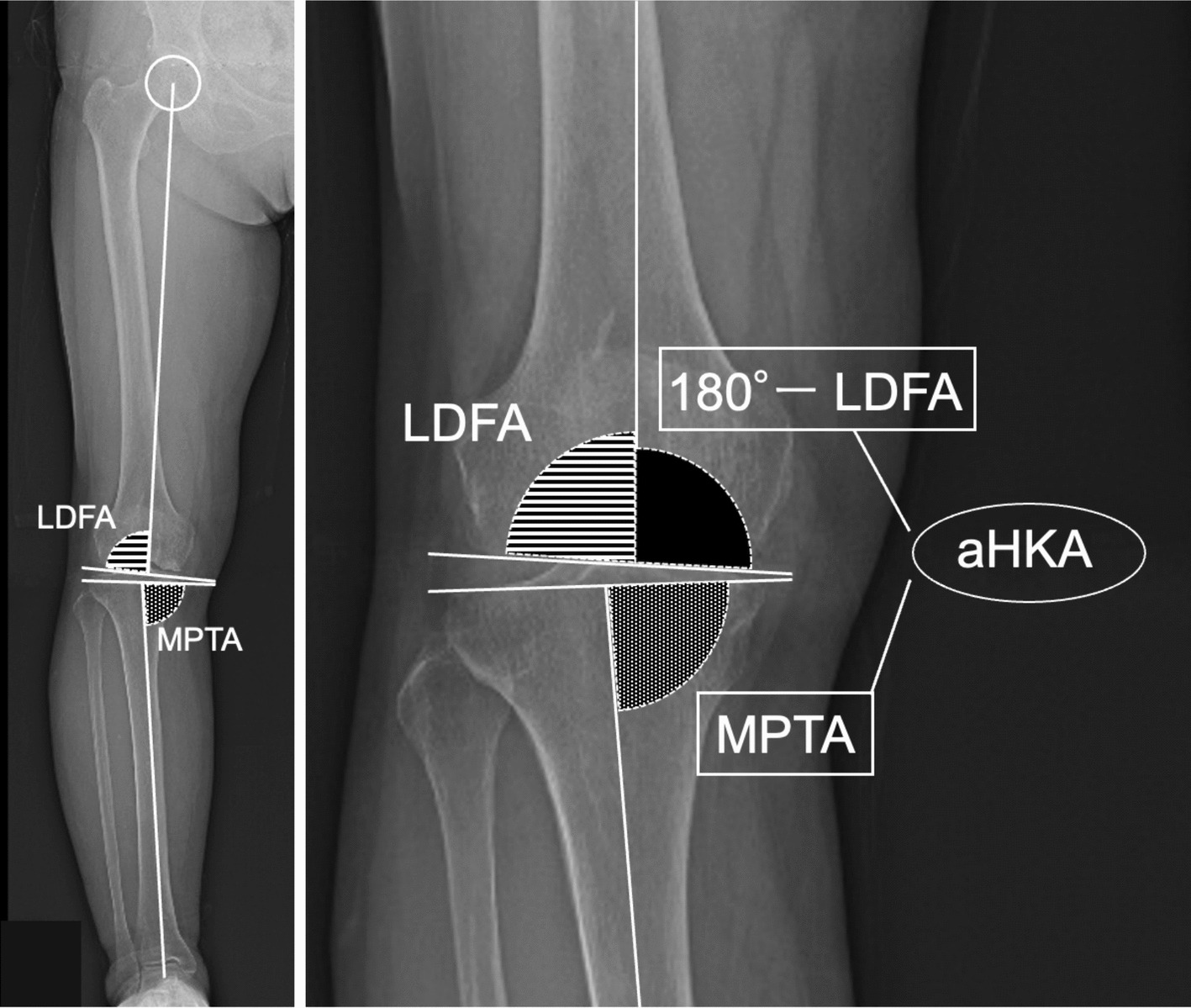
Measurement of aHKA. *aHKA was calculated as 180° − LDFA* + *MPTA*

**Fig. 2:**
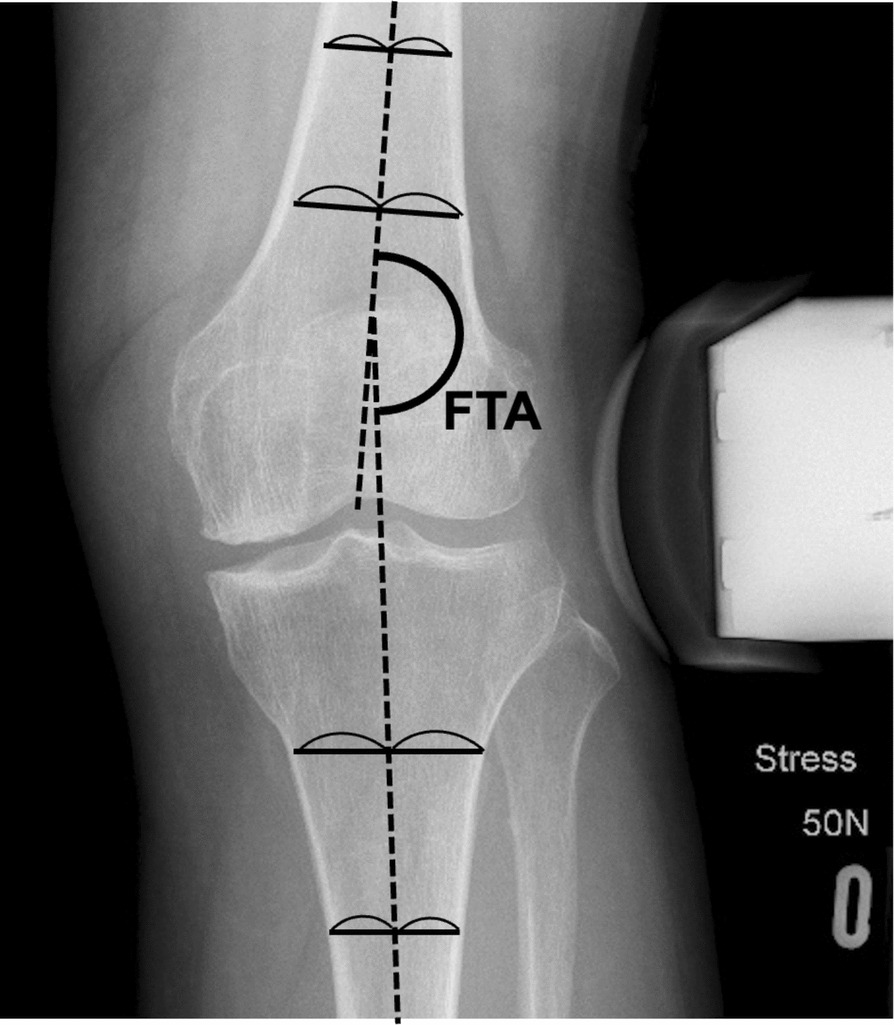
Measurement of valgus stress FTA. *FTA is defined as the lateral angle formed by the intersection of the femoral and tibial anatomical axes*

Pre- and postoperative coronal HKA were measured using long-leg standing radiographs. LDFA was determined as the lateral angle formed by the mechanical axis of the femur and a line drawn across the articular surface of the distal femur at the lowest points of the lateral and medial femoral condyles. MPTA was measured as the medial angle between the mechanical axis of the tibia and a line connecting the most distal articular contours of the midpoints of the lateral and medial plateaus. The aHKA was calculated using the formula: 180° − LDFA + MPTA (Fig. 1). Preoperative valgus stress FTA were measured in anteroposterior radiographs of the knee in full extension using a Telos SE arthrometer (Telos, Greisheim, Germany) with an 11.24 lb (50 N) force. In the femur, a line along the anatomical axis of the femur through the center of the femoral shaft was drawn using two mid-diaphyseal points above the knee. In the tibia, a line along the anatomical axis of the tibia was drawn using two mid-diaphyseal points below the knee. FTA is defined as the lateral angle formed by the intersection of the femoral and tibial anatomical axes (Fig. 2).

All data are expressed as means ± standard deviations. To evaluate the intra-observer and inter-observer reliability of the measurements for FTA, LDFA, MPTA, and HKA, two investigators independently assessed the first 10 patients on two occasions, and intra-class correlation coefficients (ICCs) were calculated. The ICCs for intra-observer reliability exceeded 0.84 (range, 0.84–1.00), while those for inter-observer reliability exceeded 0.82 (range, 0.82–0.96) across all parameters. Correlations between postoperative HKA and aHKA, as well as 360° minus the valgus stress FTA, were examined using simple linear regression analysis. Similarly, correlations between ΔHKA and (aHKA–preoperative HKA), as well as (360°–valgus stress FTA–preoperative HKA), were analysed using the same statistical method. Pearson’s correlation coefficients were calculated, and Fisher’s Z-transformation was applied to assess the significance of the difference between the two independent correlation coefficients.

Data analyses were performed using BellCurve for Excel (Social Survey Research Information Co., Ltd., Tokyo, Japan). A sample size calculation was conducted using G*Power 3 (Heinrich Heine Universität Düsseldorf, Germany). Based on the assumptions of an effect size f^2^ of 0.15, a type I error rate (α) of 0.05, and a power (1 − β) of 0.95, the required minimum sample size for the regression analysis was calculated to be 89 patients. A P value of less than 0.01 was considered statistically significant.

## Results

A total of 125 knees in 101 patients were enrolled in the study. The mean ± SD of pre- and postoperative radiological parameters, i.e., HKA, aHKA, ΔHKA, and valgus stress FTA, were presented in Table 2.

**Table 2:** Radiological parameters

Variables	Value
Preoperative HKA (°)	172.4 (range, 163.8–179.1; SD: 2.8)
Postoperative HKA (°)	177.0 (range, 170.2–183.0; SD: 2.5)
ΔHKA (°)	4.6 (range, 0.4–11.1; SD: 2.1)
aHKA (°)	177.2 (range, 172.3–182.4; SD: 2.1)
LDFA (°)	87.8 (range, 84.1–90.4; SD: 1.2)
MPTA (°)	85.0 (range, 81.1–89.1; SD: 1.9)
Valgus stress FTA (°)	176.3 (range, 170.3–184.4; SD: 2.8)

The values are given as the mean and standard deviation for continuous variables

HKA: hip–knee–ankle angle, ΔHKA: the degree of alignment correction achieved through operation, aHKA: arithmetic hip–knee–ankle angle, LDFA: lateral distal femoral angle, MPTA: medial proximal tibial angle, FTA: femorotibial angle, SD: standard deviation

**Fig. 3:**
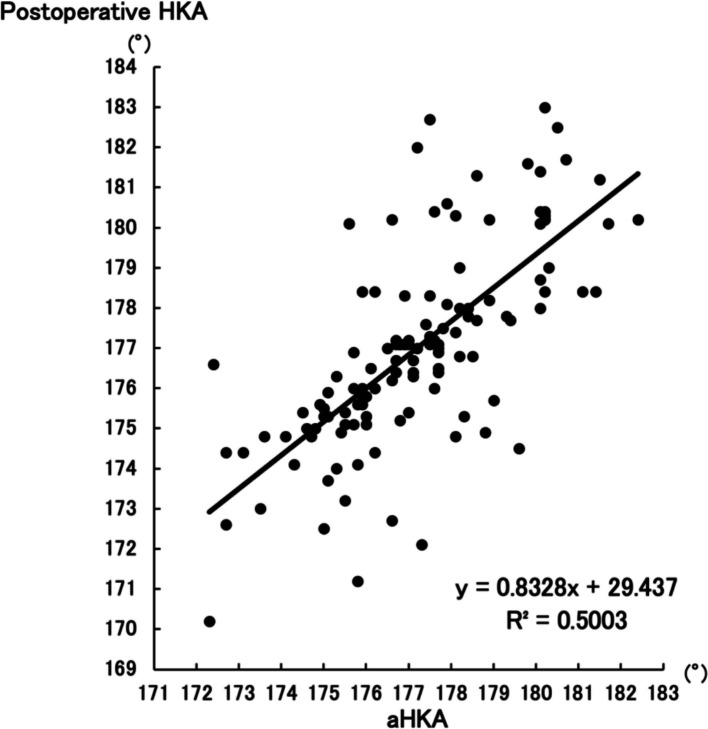
Correlation between postoperative HKA and aHKA. *The association between postoperative HKA and aHKA, with a correlation coefficient of R*^*2*^ = *0.5003 (P* < *0.001)*

**Fig. 4:**
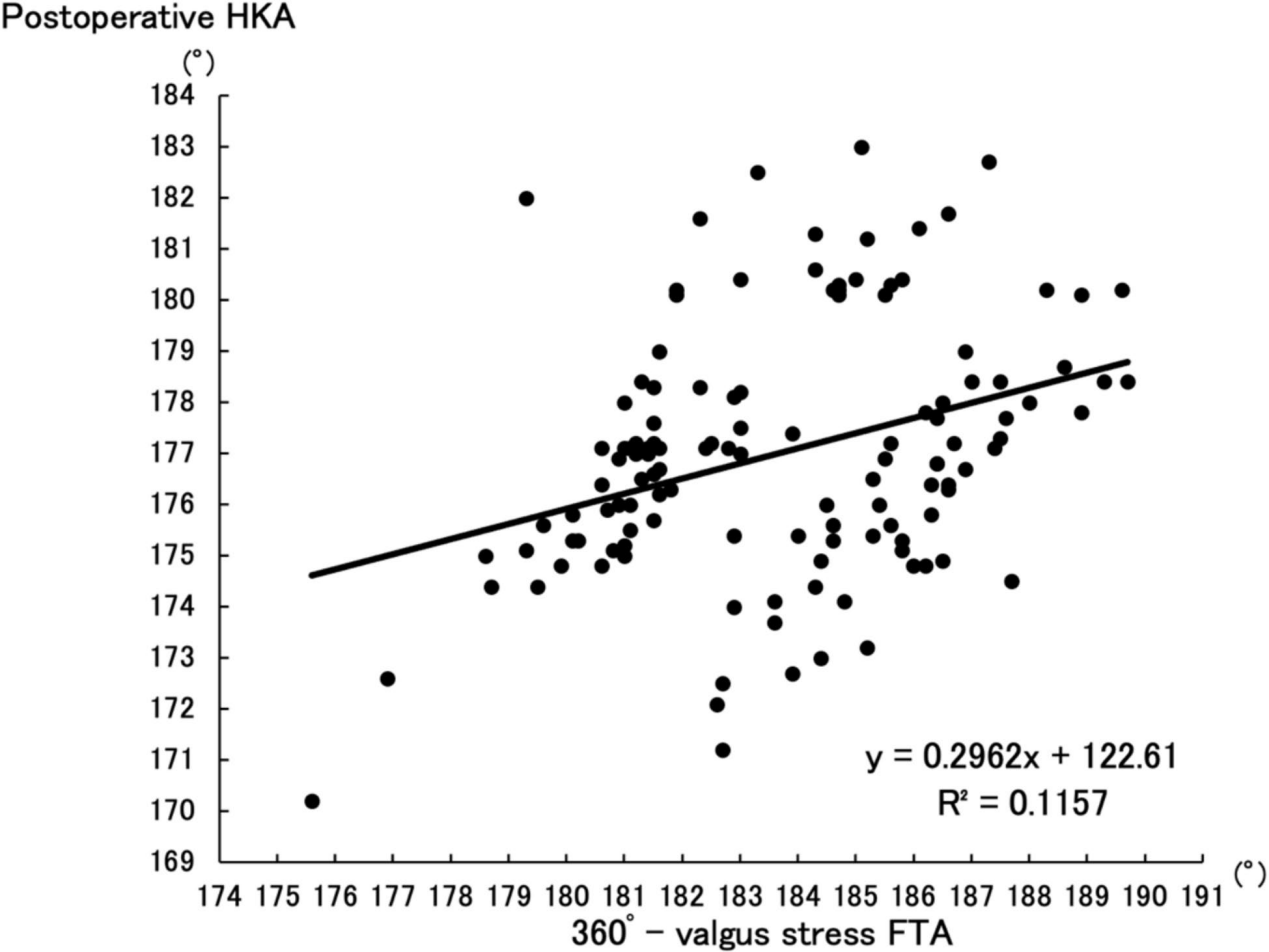
Correlation between postoperative HKA and 360°–valgus stress FTA. *The association between postoperative HKA and 360°–valgus stress FTA with a correlation coefficient of R*^*2*^ = *0.1157 (P* < *0.001)*

There was a positive correlation between postoperative HKA and aHKA (R^2^ = 0.5003, *P* < 0.001) (Fig. 3) as well as postoperative HKA and (360°–valgus stress FTA) (R^2^ = 0.1157, *P* < 0.001) (Fig. 4). To assess whether the difference in correlation coefficients was statistically significant, a Fisher’s Z-transformation was applied. The Z-score for the difference between the two independent correlations was −4.12, yielding *P* < 0.001. The results indicate that the former group demonstrates a significantly stronger correlation compared to the latter group.

**Fig. 5:**
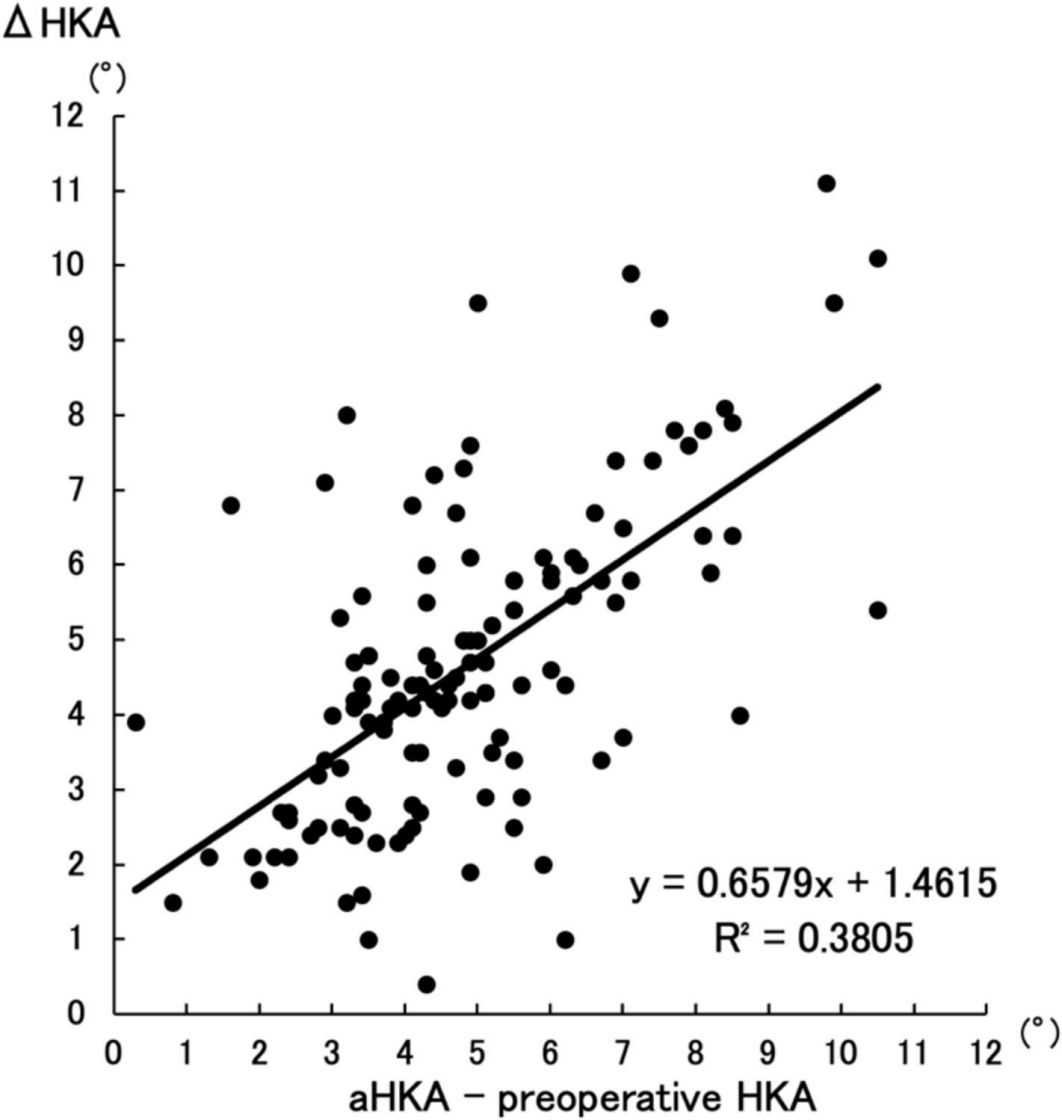
Correlation between ΔHKA and aHKA–preoperative HKA. *The association between ΔHKA and the aHKA–preoperative HKA, with a correlation coefficient of R*^*2*^ = *0.3805 (P* < *0.001)*

**Fig. 6:**
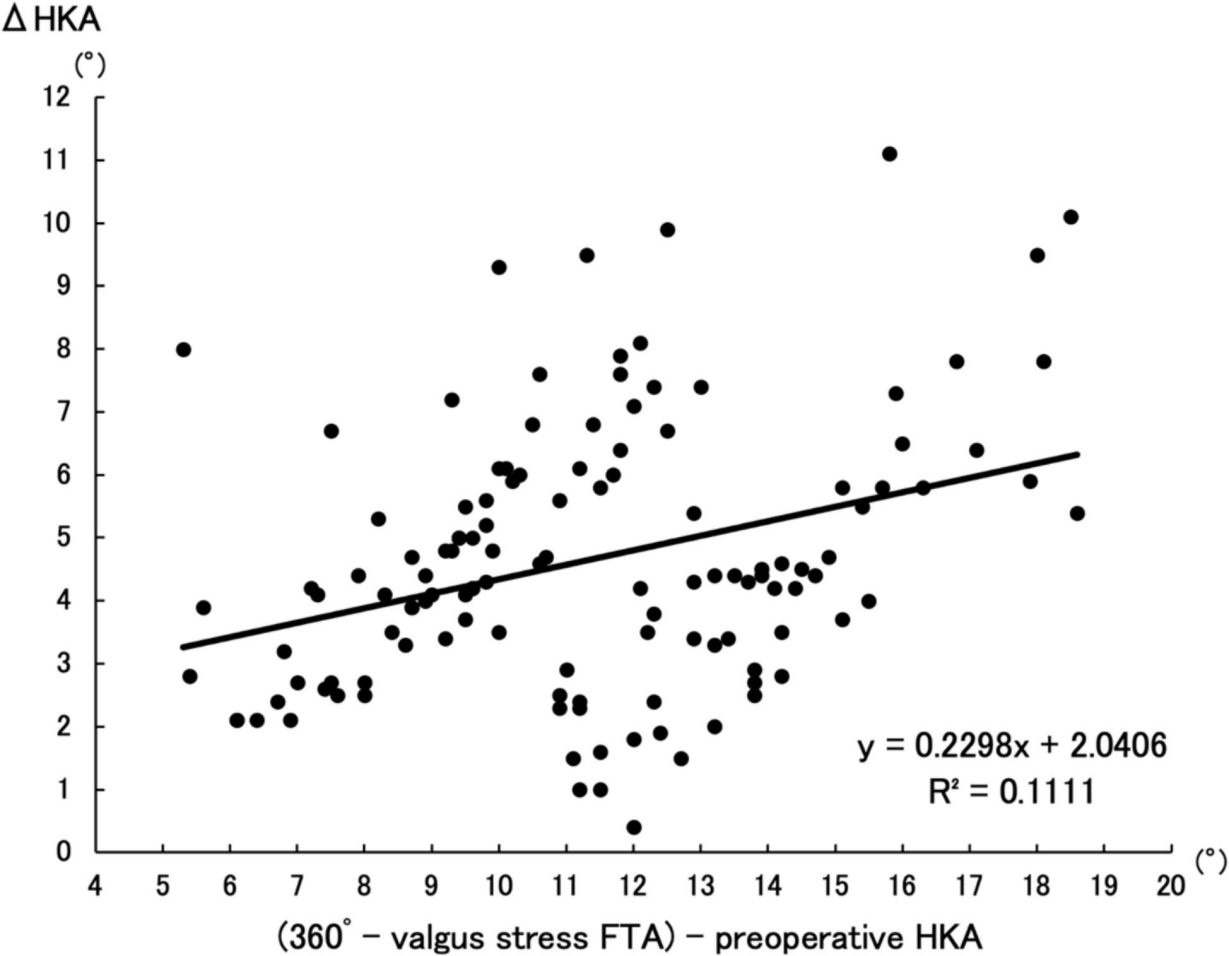
Correlation between ΔHKA and (360°–valgus stress FTA)–preoperative HKA. The association between ΔHKA and (360°–valgus stress FTA)–preoperative HKA, with a correlation coefficient of *R*^2^ = 0.1111 (*P* < 0.001)

As for ΔHKA, there was a positive correlation between ΔHKA and (aHKA–preoperative HKA) (R^2^ = 0.3805, *P* < 0.001) (Fig. 5) as well as between ΔHKA and (360°–valgus stress FTA–preoperative HKA) (*R*^2^ = 0.1111, *P* < 0.001) (Fig. 6). The Z-score for the difference between the two independent correlations was −2.92, yielding *P* = 0.0036. The results indicate that the former group demonstrates a significantly stronger correlation compared to the latter group.

## Discussion

This study demonstrated that aHKA is a significantly more reliable predictor of postoperative coronal alignment following medial UKA than valgus stress FTA. Furthermore, the difference between aHKA and preoperative HKA correlated more strongly with ΔHKA than the corresponding difference based on valgus stress FTA. These findings highlight the superiority of bone-based alignment metrics over soft tissue–dependent methods in preoperative planning for UKA.

Restoration of appropriate coronal alignment following UKA is clinically crucial. Postoperative alignment abnormalities, particularly excessive residual varus or valgus deformities, have been well documented as significant risk factors for early failure after UKA. Such malalignment can accelerate polyethylene wear, contribute to loosening of components, and precipitate the progression of OA in the contralateral, non-operated compartment [5, 21–23]. These mechanical failures ultimately compromise implant longevity and adversely affect patient outcomes. Hence, the ability to accurately predict and restore near-neutral or slightly under-corrected limb alignment is essential for achieving durable and successful UKA results. Valgus stress FTA has traditionally been used to assess the correctability of varus deformity in patients considered for UKA. However, this method is limited by its dependence on soft tissue laxity, patient cooperation, and consistency in stress application. Despite efforts to standardize the procedure using devices such as arthrometers, the results remain variable [24–26]. Additionally, FTA is derived from anatomical rather than mechanical axes, which may reduce its predictive value for true coronal alignment. In this study, the correlation between postoperative HKA and 360°–valgus stress FTA was weak (*R*^2^ = 0.1157), underscoring its limited predictive value. In contrast, aHKA is calculated solely from the mechanical alignment of the femur and tibia, using LDFA and MPTA. It is thus inherently independent of soft tissue conditions and examiner technique. The present study showed that aHKA had a markedly stronger correlation with postoperative HKA (*R*^2^ = 0.5003), suggesting that native bony morphology is a more reliable determinant of final limb alignment when standardized surgical techniques are employed. The analysis of ΔHKA further reinforced the utility of aHKA. The correlation between ΔHKA and aHKA–preoperative HKA (*R*^2^ = 0.3805) was significantly higher than that between ΔHKA and (360°–valgus stress FTA)–preoperative HKA (*R*^2^ = 0.1111). This suggests that aHKA not only predicts the postoperative alignment more accurately but also provides a more precise estimate of the expected magnitude of correction. This is particularly important in UKA, where the aim is to achieve near-neutral or slightly under-corrected alignment to preserve adjacent compartment integrity and implant longevity [27, 28].

The superiority of aHKA observed in this study supports previous reports that advocated its use in UKA planning [16, 17]. While earlier studies demonstrated its correlation with postoperative alignment, the present study is the first to directly compare aHKA with valgus stress FTA using formal statistical analysis within the same cohort. The application of Fisher’s Z-transformation provided strong statistical evidence for the superiority of aHKA in both predicting postoperative alignment and estimating alignment correction. In terms of clinical applicability, aHKA offers multiple advantages. It can be calculated from standard long-leg standing radiographs, without requiring additional stress imaging or equipment. It avoids the variability associated with soft tissue behavior, pain, or muscle tone. As a result, aHKA is especially useful in cases where patients cannot tolerate valgus stress radiography or when reproducibility is a concern. In modern practice, where digital templating and data-driven planning are increasingly emphasized, aHKA represents a simple, objective, and scalable tool for preoperative assessment. While the traditional valgus stress FTA cutoff of 180° has served as a practical guideline, its poor predictive performance in this and previous studies calls its continued use into question [8, 12]. In contrast, defining a clinically meaningful aHKA threshold to guide patient selection for UKA may provide a more robust, standardized, and evidence-based criterion. Such a threshold would reflect the patient’s constitutional bony alignment and could better predict whether correction to a safe postoperative alignment can be achieved. Further large-scale prospective studies are warranted to validate such cutoffs and correlate them with long-term clinical outcomes.

Several limitations of this study should be acknowledged. First, it was a retrospective analysis conducted at a single institution, which may limit generalizability. Second, all procedures utilized an accelerometer-based navigation system, which likely enhanced the precision of bone cuts and alignment correction; results may differ in centers employing conventional instrumentation. Third, the study cohort consisted exclusively of patients undergoing fixed-bearing medial UKA, and findings may not apply to mobile-bearing designs, where biomechanical goals and alignment strategies differ. Fourth, although aHKA showed stronger correlations with radiographic outcomes, it should not be considered the sole determinant of successful alignment restoration. Intraoperative factors such as ligament balancing, femoral rotation, and tibial slope also influence the final alignment and clinical results. Finally, this study focused on radiographic parameters without evaluating functional or patient-reported outcomes; therefore, the relationship between aHKA-based planning and long-term clinical success remains to be elucidated.

## Conclusion

In conclusion, aHKA demonstrated a significantly stronger correlation with postoperative HKA, and the difference between aHKA and preoperative HKA showed a significantly stronger correlation with ΔHKA, compared to corresponding FTA-based measures. As an objective and reproducible metric based on bone morphology, aHKA offers clear advantages in the preoperative evaluation and planning of medial UKA. These findings support the broader adoption of aHKA in clinical practice and may reduce the need for stress radiographs in routine UKA assessment.

## Data Availability

No datasets were generated or analysed during the current study.
